# Heavy metal toxicity in poultry: a comprehensive review

**DOI:** 10.3389/fvets.2023.1161354

**Published:** 2023-06-29

**Authors:** Abdullah S. M. Aljohani

**Affiliations:** Department of Veterinary Medicine, College of Agriculture and Veterinary Medicine, Qassim University, Buraidah, Saudi Arabia

**Keywords:** poultry, lead, cadmium, environmental pollution, disease, toxicity

## Abstract

Arsenic (As), lead (Pb), cadmium (Cd), and mercury (Hg) have been recognized as most toxic heavy metals that are continuously released into the environment, both from natural sources and from anthropogenic production of fertilizers, industrial activities, and waste disposal. Therefore, As, Cd, Hg, and Pb are found in increasing concentrations in bodies of water, fodder, feed, and in the tissues of livestock, including poultry, in the surroundings of industrial areas, leading to metabolic, structural, and functional abnormalities in various organs in all animals. In poultry, bioaccumulation of As, Pb, Cd, and Hg occurs in many organs (mainly in the kidneys, liver, reproductive organs, and lungs) as a result of continuous exposure to heavy metals. Consumption of Cd lowers the efficiency of feed conversion, egg production, and growth in poultry. Chronic exposure to As, Pb, Cd, and Hg at low doses can change the microscopic structure of tissues (mainly in the brain, liver, kidneys, and reproductive organs) as a result of the increased content of these heavy metals in these tissues. Histopathological changes occurring in the kidneys, liver, and reproductive organs are reflected in their negative impact on enzyme activity and serum biochemical parameters. Metal toxicity is determined by route of exposure, length of exposure, and absorbed dosage, whether chronic and acute. This review presents a discussion of bioaccumulation of As, Cd, Pb, and Hg in poultry and the associated histopathological changes and toxic concentrations in different tissues.

## Introduction

**Figure 1:**
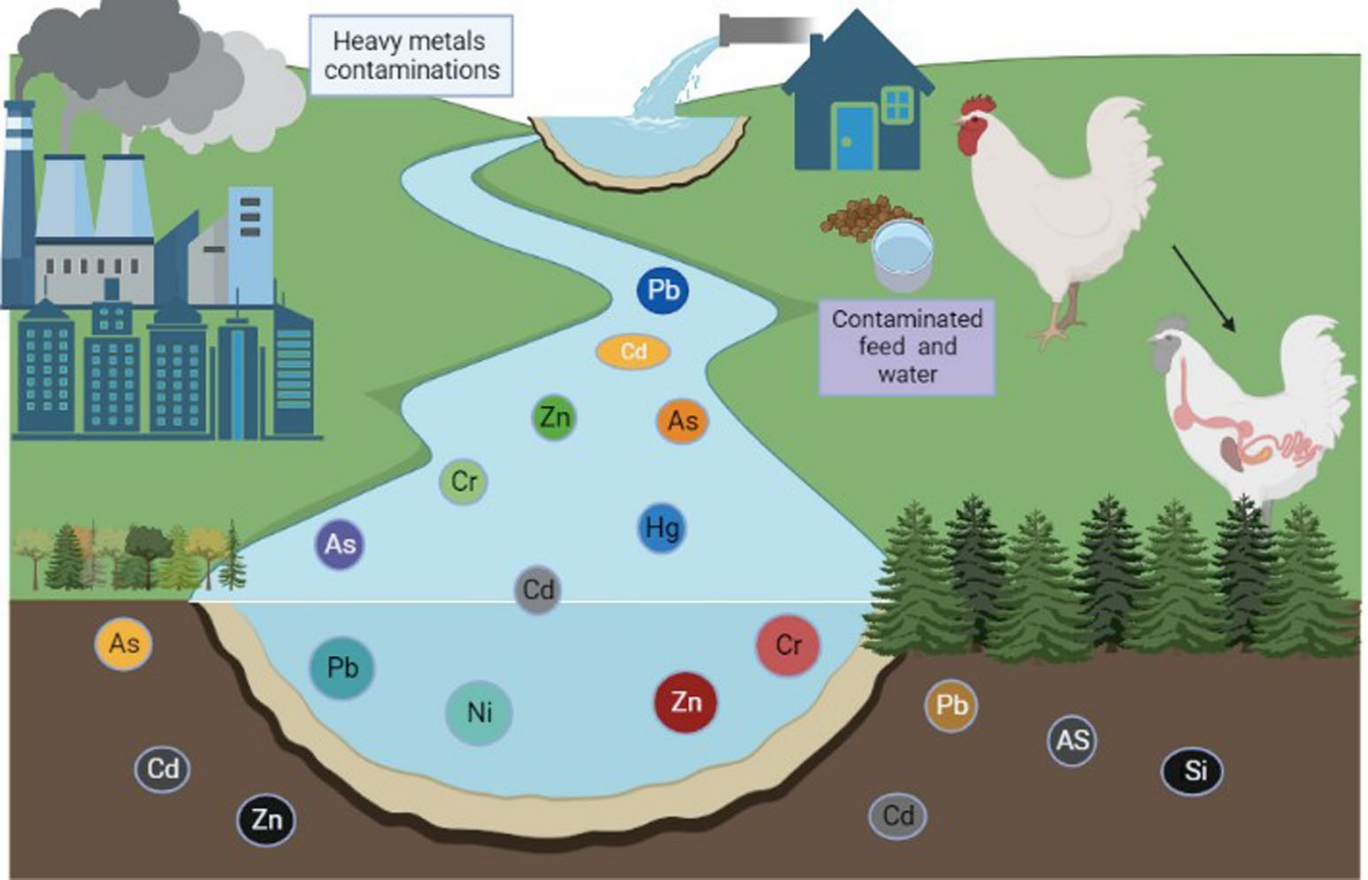
Heavy metal contamination in poultry (produced using BioRender).

**Figure 2:**
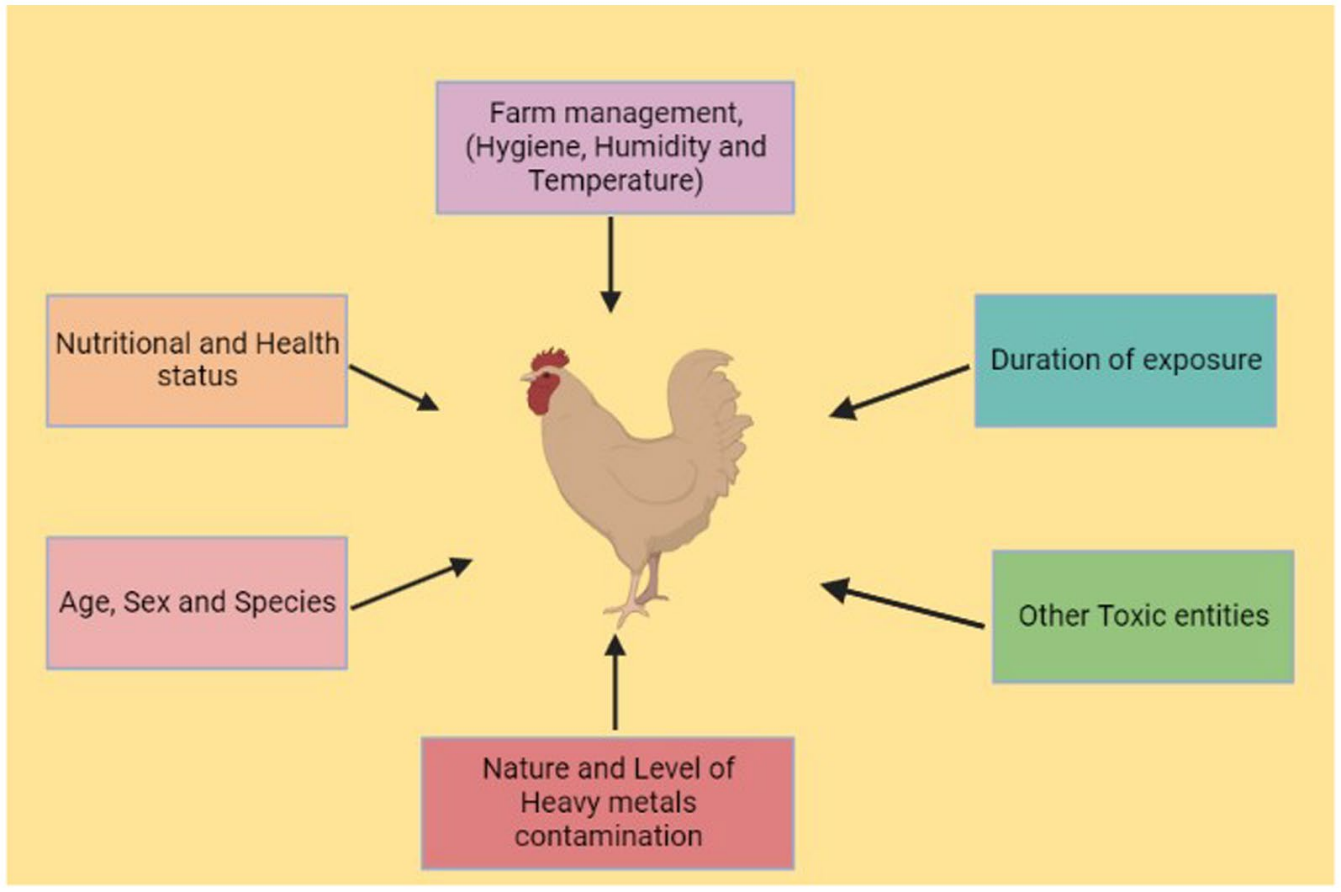
Factors affecting poultry production (produced using BioRender).

Heavy metals are members of the class of metalloids and metals with an atomic density greater than or equal to 4,000 kg/m3 (1). Animals can absorb environmental elements and metals from the air, water, sediment, and food (2, 3). Heavy metals are among the main contaminants of our food supply, and heavy metal contamination is a serious issue for our ecosystem (4). Heavy metal contamination is pervasive throughout the world, especially in areas close to urban regions and industrial zones (5). Zinc (Zn), iron (Fe), copper (Cu), and selenium (Se) are essential metals that have specific functions in regulating body metabolism (6, 7). In contrast, toxic elements such as lead (Pb), chromium (Cr), mercury (Hg), nickel (Ni), and cadmium (Cd) are typically associated with contamination and can have hazardous effects on living organisms when specific concentrations are exceeded (8, 9). Nonessential elements have no known specific function in the body but are also not assumed to be toxic to any significant degree (9). Trace amounts of some heavy metals, such as Cd, Pb, As, Cr, Hg, and Ni, can be found in water, poultry, fish, and birds (4, 10). Prolonged exposure to these heavy metals, even at low doses, can have severe negative effects on both animal and human health (11), and the buildup of heavy metals in the environment and biosphere is considered to be a biohazard (12, 13). Metal pollutants are already present in the atmosphere, but may become more prevalent as a result of pollution and industrial activity (Figure 1) (14). In particular, expanding patterns of anthropogenic activity (including industrialization, mining, the use of chemical fertilizers and pesticides, unrestricted sewage discharge, and extensive groundwater irrigation) have accelerated the spread of heavy metals (15, 16). A wide range of factors contribute to the presence of toxic metals in agricultural soils, including air deposition, sewage irrigation, agrochemicals, and animal and bird manure (17–19). Agricultural soil contains heavy metals that have a prolonged residence time (often many decades) and sustained bioavailability (Figure 2) (20) due to the toxicity of heavy metals at low levels of exposure. Many of these toxic metals can pose serious ecological threats to animals (21, 22), even threatening the health of poultry and animals through food chain transmission and accumulation (23).

After air deposition, the application of poultry and animal manure is the main source of the majority of heavy metals found in agricultural soil (24–26). The use of poultry and livestock manure in certain ways has contributed to the accumulation of several heavy metals (including Cd and Hg) in cultivated fields over the past decade (27–29).

**Figure 3:**
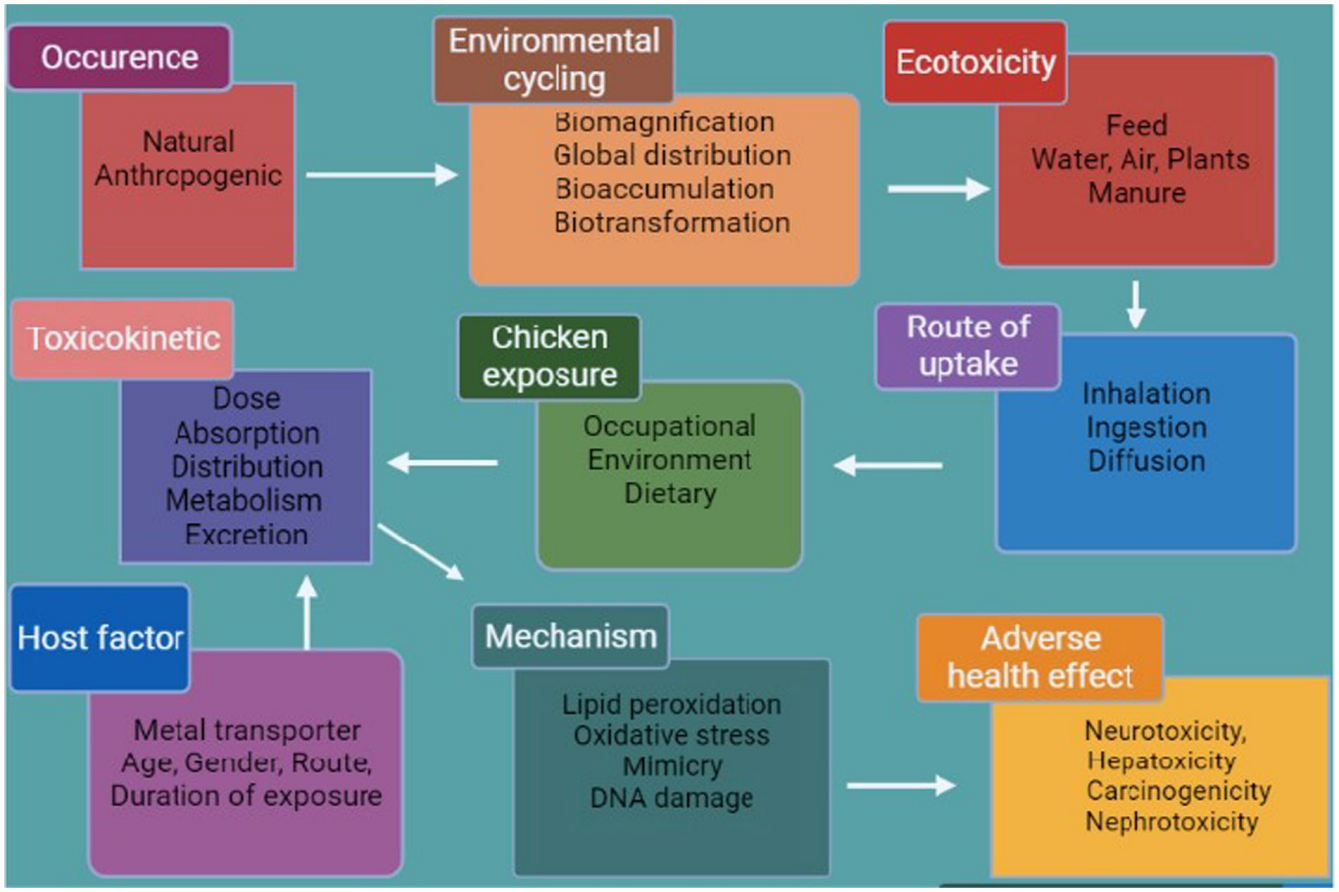
Exposure of poultry to heavy metals (produced using BioRender).

Pb and Cd are the most poisonous of the most common heavy metals to accumulate in the food chain. Following absorption, these are predominantly dispersed across several tissues, mainly the kidneys and liver (30, 31). The accumulation of a high level of heavy metals triggers a variety of deadly symptoms, such as reproductive issues and hepato–renal dysfunction (32). Pb is a neurotoxin that can impair metabolism and exert negative effects on the neurological, gastrointestinal, and renal systems, as well as hemopoiesis and renal function (33). Pb exposure can block heme synthesis and harm the brain and kidney systems (Figure 3) (34). Diet is a source of Cd contamination; this arises from a variety of food sources and from the environment and is passed to animals through the food chain (35), causing hypertension, kidney dysfunction, and damage to the lungs and liver as well as pulmonary and hepatocellular tissue (36).

The liver and kidneys are crucial for detoxification and the excretion of hazardous substances in both humans and animals (37). Organs sustain the most harm when there is an overabundance of poisonous substances in feed (38), and this depends on the type of feed consumed. In terms of specific metals, As is stored in animal tissues and can cause nausea, headache, and severe gut irritation (39, 40). Like other metals, Cu impairs liver, kidney, and brain functions at high doses and can cause hemolytic crisis (41).

Poultry farming is one of the most important food-producing industries in the world (42), and poultry is the main source of protein for millions of people across the world (43). In 2019, worldwide egg production reached 83 Mt., a 63% increase since 2000, and poultry meat represented approximately 40% of worldwide meat production, highlighting its significance as the most widely produced meat globally (44). Numerous toxic metals are found as trace components and used as feed additives in poultry feed (45). The hazardous effects of heavy metals on poultry include loss of weight, organ failure, and death (46, 47). Metal toxicity is determined by route of exposure, length of exposure, and absorbed dosage, whether chronic or acute. The aim of this review is to present a comprehensive account of the mechanisms of heavy metal toxicity, its effects, and the histopathological changes that occur in different tissues in poultry under exposure to heavy metals.

## Sources of heavy metal transmission and their impact on poultry

### Sources of cadmium transmission

Cd is a significant environmental contaminant that is continuously released into the environment from industrial and natural sources (48, 49). Along with numerous other pollutants, Cd is a contaminant of the atmosphere with two types of sources, anthropogenic and natural. The contribution of anthropogenic sources is three to 10 times more dangerous than that of natural sources (5, 50). The main natural sources include forest fires, movement *via* wind-borne soil, and volcanic eruptions (51, 52). The smelting of Cu and Ni, the burning of fossil fuels, the production of phosphate fertilizers from rocks containing different levels of Cd, and the usage of sewage sludge in soil are all examples of anthropogenic sources. Cd is dispersed into soil and water, where it accumulates in biogenic species through food chains and presents a threat to poultry health. Cd can enter the bodies of poultry animals to a small extent *via* food and drinking water (53).

### Effects of cadmium on poultry

Cd is transported to target tissues, where it accumulates, after binding to metallothionine in the bloodstream (54). Cd has teratogenic consequences in various animals, including chickens, such as appendage deformities, ear abnormalities, and gastrointestinal problems (55, 56). Additionally, non-hypertrophic emphysema, osteoporosis, persistent rhinitis, anemia, and eosinophilia can all result from Cd exposure (57, 58). When the amount of Cd in the blood exceeds the metallothionine ability to bind it, free Cd triggers the production of free radicals and lipid peroxidases, which harm the liver and kidneys (59). Ingestion of Cd at a high rate results in a reduction in egg production by poultry as a result of histopathological damage, reducing feed intake and increasing sensitivity to stress (60, 61). Furthermore, absorption of Cd in the digestive tract increases deficiencies of minerals such as Fe and Ca normally obtained via the diet (62). In addition to increasing bioaccumulation in tissues, exposure to Cd in poultry also transfers Cd to eggs. Cd exposure may lower the protein concentration needed for absorption and transport, and thus decreases excretory activity in the oviduct in poultry (56).

### Sources of lead transmission

Animals are routinely exposed to Pb, which is one of the greatest environmental poisons in industrialized areas of the world (63). Pb is a naturally occurring element in the inner layer of the earth’s crust; it enters the environment in various ways, including the burning of gasoline (the primary source of Pb exposure), plant fuel, drinking water, recycled material, dust, cosmetics, and lead-based paints (64, 65). Pb poisoning, which is particularly prevalent in animals, can be brought on by a variety of environmental variables, including industrial pollutants, agricultural practices, use of automobiles, and contaminated feed and soil (66, 67). Pb ingested orally is only slightly absorbed by the animals; however, after constant exposure at a low level, due to the relatively slow rate of Pb removal, a hazardous level of Pb can accumulate in tissues (68). When Pb comes into contact with air, food, and drink, it has an impact on all biological systems, including that of poultry (69).

### Effects of lead on poultry

Pb has the capacity to cause oxidative stress and serves as a catalyst for oxidative processes of biological molecules by generating free radicals (70). Depending on the degree of exposure, the negative consequences of Pb can range from minor physiological or biochemical abnormalities to significant pathologic illnesses, in which various organs and systems may be harmed or their functions altered (71). Pb acetate in subclinical amounts reduces the sensitivity of chickens to endotoxins. Pb has the potential to deactivate antibodies, thus impairing the resistance of poultry to infectious illness (72). Pb poisoning also reduces lysosome activity and is involved in phagocytic activity of polymorphonuclear leukocytes (73). Finally, Pb obstructs the actions of many antioxidant defenses; low antioxidant levels may damage various organ systems, including the nervous system, the liver, the kidneys, and the reproductive system (74). In severe cases, Pb toxicity has also been shown to cause death in poultry (75).

### Sources of arsenic transmission

As is a chemical found in the environment that has a significant impact on the health of animals, including poultry (76). As can be found in trivalent, pentavalent, organic, and inorganic forms and can combine with variety of elements, such as S, H, O, Pb, and Cu (31, 77). Similar to animal exposure more generally, poultry in As-affected areas are exposed to dangerous level of the toxic metal (78). As is a source of toxicity and is typically present in fluids used to spray animals to control ectoparasites (3, 79). Feed ingredients, contaminated drinking water, vegetables, grasses, plants, and atmospheric emissions are sources of As contamination (80), with the first four mentioned being the main sources of As (81).

### Effects of arsenic On poultry

The role of arsenic in poultry nutrition is heavily disputed; it is highly hazardous even in very low quantities in food (82). In poultry, acute As poisoning causes circulatory collapse, stomach pain, excessive salivation, hypothermia, watery diarrhea, and death (83, 84). Symptoms of long-term exposure to As at low concentrations in poultry include chronic indigestion, stomach cramps, and skin discoloration (85, 86). Long-term consequences can include gangrene-like sores, carcinoma of the skin, liver, kidneys, and lungs, and cancer (87, 88). The liver is typically thought to be the primary organ involved in the metabolism of As (89). As can block the action of intracellular enzymes and may impact acetyl-CoA synthesis, glutathione (GSH) synthesis, fatty acid oxidation, glucose uptake, and gluconeogenesis (90). One of the most frequently recognized explanations for As-induced toxicity is oxidative stress: oxidative stress brought on by As-induced liver damage results in the production of reactive oxygen species (ROS) (91). Despite the fact that As cannot directly cause DNA damage, it still has an impact on the enzymes involved in DNA repair and the energy pathway of cells. Finally, As causes oxidative damage in the skeletal muscles, liver, and kidneys in chickens (92).

### Sources of mercury transmission

Hg is one of the most potent neurotoxins, and it has a range of negative health effects on both humans and animals (93). Hg is considered to be a significant environmental pollutant, along with other non-essential trace metals, because of its high toxicity and capacity for biomagnification and bioaccumulation (94). Methyl mercury is known to be the most dangerous form, but Hg (II) is more frequently and abundantly present in the environment and has the potential to exert extremely negative effects on poultry (95). Hg can exist in environment in the form of metal divalent, monovalent, dimethyl mercury, and methyl mercury. Inorganic mercury salts and organic mercury compounds make up the majority of the mercury found in water, soil, sediments, plants, and animals (96, 97). The main sources of Hg include the paper industry, chemical industry, paint industry, insecticides, and fungicides, as well as geothermal steam used to generate electricity (98). Hg was originally utilized in medicine, but this therapeutic use was halted due to its severe toxic effects in both people and animals (99).

### Effects of mercury on poultry

Hg is recognized as a toxic chemical that can cause devastating effects in poultry, such as kidney and liver damage, even at a very low level of exposure (100). Toxic concentrations of Hg are dangerous for poultry, with symptoms including development of anemia and depressed growth rate. Young growing chickens are typically more susceptible to the toxic effects of chronic Hg exposure than adults (101, 102). The production of oxidative stress, suppression of nitric oxide, and the disruption of cytokine profiles are the main mechanisms of Hg-induced toxicity in immune cells (103). To assess the effects of Hg exposure on the immune system in poultry, activated immunity should be considered, as this is more important in vulnerability to diseases. Hg exposure can damage tissues and organs, and it is absorbed and distributed in the liver and kidneys in poultry (104, 105).

### Worldwide reports on heavy metal toxicity in poultry

Metal toxicity has been observed in many living organisms, but our main focus here is on poultry. It has been found that metal toxicity is highly prevalent in poultry worldwide, as outlined in Table 1. Various heavy metals have been examined in different studies, among which one study has measured the concentrations of Pb, Cd, Ni, Hg, Fe, Zn, Mg, and Cu in the kidneys, spleen, and liver of poultry from Manisa, Turkiye. Concentrations of heavy metals can be determined using atomic absorption spectrophotometry (106, 128). The highest concentration of Cu was observed in the liver, at 3.7 mg/kg, and the lowest level in the spleen, at 1.99 mg/kg (129). For Pb, the highest concentration was observed in the kidney, at 0.103 mg/kg, and the lowest level in the liver of chickens, at 0.065 mg/kg. The concentrations of Pb and Hg in the liver in chicken were found to be 0.102 and 0.053 mg/kg, respectively (106).

**Table 1:** Metal toxicity observed in different organs in poultry.

Metal	Organ	Normal range	Toxic concentration	Country	References
Cadmium	Liver	0.039 mg/kg	0.050 mg/kg	Türkiye	(106)
Cadmium	Kidney	0.011 mg/kg	0.075 mg/kg	Türkiye	(106)
Cadmium	Spleen	0.011 mg/kg	0.084 mg/kg	Türkiye	(106)
Cadmium	Liver	0.16 mg/kg	0.627 mg/kg	Malaysia	(107)
Cadmium	Meat	19.67 μg/g	26.7 μg/g	Malaysia	(107)
Cadmium	Liver	0.15 μg/g	0.221 μg/g	Malaysia	(107)
Cadmium	Liver	0.04 mg/kg	0.095 mg/kg	Iraq	(108)
Cadmium	Kidney	0.018 mg/g	0.019 mg/g	Nigeria	(109)
Cadmium	Liver	0.03 mg/g	0.04 mg/g	Nigeria	(109)
Cadmium	Breastmeat	0.005 mg/g	0.5 mg/g	Nigeria	(109)
Cadmium	Liver	0.138 mg/kg	1.213 mg/kg	Pakistan	(110)
Cadmium	Meat	0.075 mg/kg	1.15 mg/kg	Pakistan	(110)
Cadmium	Egg	0.388 ppm	19 ppm	Saudi Arabia	(111)
Cadmium	Liver	0.137 ppm	19 ppm	Saudi Arabia	(111)
Cadmium	Kidney	1.03 mg/kg	7.73 mg/kg	China	(112)
Cadmium	Liver	4.69 mg/kg	20.4 mg/kg	China	(112)
Cadmium	Muscle	0.02 mg/kg	0.08 mg/kg	China	(112)
Cadmium	Liver	0.095 mg/kg	0.159 mg/kg	Iraq	(113)
Cadmium	Liver	0.37 mg/kg	0.627 mg/k	Iran	(114)
Cadmium	Liver	0.29 mg/kg	0.3 mg/kg	Nigeria	(115)
Cadmium	Meat	0.040 μg/g	0.94 μg/g	India	(116)
Cadmium	Liver	0.01 mg/kg	0.29 mg/kg	Saudi Arabia	(117)
Cadmium	Kidney	0.0053 ppm	0.1324 ppm	Iraq	(118)
Cadmium	Meat	0.05 ppm	0.0953 ppm	Iraq	(118)
Cadmium	Meat	0.097 ppm	12–40 ppm	Saudi Arabia	(119)
Cadmium	Liver	0.07 mg/kg	0.3 mg/kg	Iraq	(120)
Cadmium	Brain	0.99 mg/kg	2.493 mg/kg	Bangladesh	(121)
Cadmium	Liver	0.998 mg/kg	2.489 mg/kg	Bangladesh	(121)
Cadmium	Kidney	0.15 mg/kg	62.93 mg/kg	Tunisia	(122)
Cadmium	Liver	0.14 mg/kg	7.80 mg/kg	Tunisia	(122)
Cadmium	Muscle	0.009 mg/kg	0.15 mg/kg	Tunisia	(122)
Cadmium	Egg	2.99 μg/kg	65.28 μg/kg	Thailand	(123)
Cadmium	Blood	1.50 μg/kg	6.18 μg/kg	Thailand	(123)
Lead	Liver	0.065 mg/kg	0.065 mg/kg	Türkiye	(106)
Lead	Kidney	0.064 mg/kg	0.092 mg/kg	Türkiye	(106)
Lead	Spleen	0.103 mg/kg	0.082 mg/kg	Türkiye	(106)
Lead	Liver	0.35 mg/kg	0.171 mg/kg	Malaysia	(107)
Lead	Liver	0.35 μg/g	0.375 μg/g	Malaysia	(107)
Lead	Liver	1.29 mg/kg	3.4 mg/kg	Iraq	(124)
Lead	Liver	2.7 mg/kg	2.9 mg/kg	Pakistan	(110)
Lead	Meat	2.15 mg/kg	2.275 mg/kg	Pakistan	(110)
Lead	Kidney	0.22–1.22 mg/kg	0.52–2.61 mg/kg	China	(112)
Lead	Liver	0.30 mg/kg	0.85 mg/kg	China	(112)
Lead	Muscle	0.04 mg/kg	0.30 mg/kg	China	(112)
Lead	Liver	0.095 mg/kg	0.1 mg/kg	Iraq	(113)
Lead	Liver	3.79 mg/kg	4.6 mg/kg	Iran	(114)
Lead	Liver	2.56 ppm	107.1 ppm	Saudi Arabia	(125)
Lead	Meat	9.21 ppm	107.1 ppm	Saudi Arabia	(126)
Lead	Liver	0.17 mg/kg	0.28 mg/kg	Nigeria	(115)
Lead	Meat	0.030 μg/g	1.91 μg/g	India	(116)
Lead	Liver	0.04 μg/g	2.04 μg/g	India	(116)
Lead	Kidney	0.05 μg/g	2.48 μg/g	India	(116)
Lead	Liver	0.14 mg/kg	0.171 mg/k	Saudi Arabia	(117)
Lead	Meat	0.001 ppm	0.0953 ppm	Iraq	(118)
Lead	Meat	2.09 ppm	10 ppm	Saudi Arabia	(119)
Lead	Liver	0.28 mg/kg	0.10 mg/kg	Iraq	(120)
Lead	Brain	1.306 mg/kg	8.548 mg/kg	Bangladesh	(121)
Lead	Liver	1.849 mg/kg	9.008 mg/kg	Bangladesh	(121)
Lead	Kidney	0.17 mg/kg	36.73 mg/kg	Tunisia	(122)
Lead	Egg	29.85 μg/kg	102.86 μg/kg	Thailand	(123)
Lead	Blood	7.57 μg/kg	77.53 μg/kg	Thailand	(123)
Mercury	Liver	0.039 mg/kg	0.084 mg/kg	Türkiye	(106)
Mercury	Kidney	0.037 mg/kg	0.075 mg/kg	Türkiye	(106)
Mercury	Spleen	0.009 mg/kg	0.014 mg/kg	Türkiye	(106)
Mercury	Liver	0.11 mg/kg	0.152 mg/kg	Iraq	(120)
Mercury	Egg	6.60 μg/kg	33.10 μg/kg	Thailand	(123)
Mercury	Blood	0.29 μg/kg	3.07 μg/kg	Thailand	(123)
Arsenic	Liver	0.1 μg/g	0.5 μg/g	Malaysia	(107)
Arsenic	Kidney	0.012 mg/g	0.036 mg/g	Nigeria	(109)
Arsenic	Liver	0.003 mg/g	0.004 mg/g	Nigeria	(109)
Arsenic	Breast Meat	0.080 mg/g	0.077 mg/g	Nigeria	(109)
Arsenic	Egg	0.00071 ppm	1.8 ppm	Saudi Arabia	(127)
Arsenic	Liver	0.0003 ppm	1.8 ppm	Saudi Arabia	(127)
Arsenic	Meat	2.76 ppm	100 ppm	Saudi Arabia	(119)

In another study, Cd, Zn, and Pb concentrations in poultry were measured in a mining area of China. In chickens, a low Pb concentration of 0.52 mg/kg was observed in the muscles (130) and a high Pb concentration of 0.63–0.73 mg/kg in the liver. Pb has been responsible for acute poisoning in poultry and has adverse effects on poultry health (131). Descending levels of concentration of Cd in chicken were observed in the liver, kidney, and muscles. In a separate study, a low Cd concentration of 4.64 mg/kg was observed in the kidneys and a high Cd concentration of 9.36 mg/kg was observed in the liver in poultry (112). A kidney: liver Cd ratio greater than 1 is an indicator of acute poisoning, whereas a ratio less than 1 indicates a lower level of poisoning (132). The highest concentrations of Zn and Cd were observed in kidneys and liver in poultry, which are known to be specific target organs for bioaccumulation of toxic metals (112).

The concentrations of several heavy metals (Zn, Cd, and Pb) were assessed in the liver, kidney, heart, and meat of chickens acquired from Kohat market, Pakistan, using a PerkinElmer PinAAcle™ 900 T atomic absorption spectrophotometer (110, 133). Concentrations of Cd in the range of 0.075 ± 0.010 to 15.763 ± 0.012 mg/kg were observed in the kidneys and liver of chickens, while concentrations of Pb in the range of 1.85 ± 0.007 to 11.838 ± 0.005 mg/kg were observed in kidneys and liver (110). It was discovered that chicken meat contained the lowest levels of concentration of these metals, while the kidneys and liver contained the most significant quantities.

In another study, heavy metals Pb, As, and Cd were measured in the liver, kidneys, and breast meat of chicken in Nigeria. An As concentration of 0.0802 ± 0.021 mg/g was observed in the breast meat and 0.0037 ± 0.018 mg/g in the liver. Cd concentrations of 0.019 ± 0.001 mg/g and 0.003 ± 0.001 mg/g were observed in the kidneys and liver, respectively (109). These results indicated that the concentration of As was higher in the breast meat and lower in the liver. The concentration of Cd was higher in the kidneys and lower in the breast meat of chickens, and Pb was not detected in samples of chicken (109).

The concentrations of heavy metals such as Cd and Pb were assessed in the liver, kidneys, and meat of chickens from an industrial area of India. The highest levels of Cd and Pb in tissues and muscles have been determined in the kidneys. Cd concentrations of 2.02 μg/g and 1.86 μg/g were observed in the kidneys and liver, respectively, in poultry (116). The findings showed that chickens in areas with toxic metal exposure may exhibit pathological lesions in various tissues as a result of heavy metal accumulation (116). As a result, eating chicken meat from the commercially exposed area may present a potential health risk.

An additional study was conducted to determine concentrations of the heavy metals Pb, Ni, and Cd in the brain and liver of poultry in Dhaka, Bangladesh, using atomic absorption spectrometry. Zn concentrations of 68.267 mg/kg and 53.778 mg/kg were observed in the liver and brain, respectively, in broiler chickens; concentrations of 348.52 mg/kg and 619.648 mg/kg were observed in liver and brain, respectively, in domestic chickens. Pb concentrations of 2.397 mg/kg in the liver and 4.141 mg/kg in the brain were observed in broiler chickens; 5.190 mg/kg in the liver and 9.008 mg/kg in the brain were observed in domestic chickens. Finally, Cd concentrations of 2.48 mg/kg in the liver and 2.493 mg/kg in the brain were observed in broiler chickens; 2.498 mg/kg in the liver and 0.999 mg/kg in the brain were observed in domestic chickens (121). These concentrations of heavy metals observed in poultry exceeded the recommended values of the WHO/FAO. A high Zn concentration of 619.648 mg/kg was observed in the brain and a low Zn concentration of 32.430 mg/kg in the liver in poultry (134).

In another study, chicken liver samples were obtained from markets in Erbil, Iraq, and inductively coupled plasma optical emission spectrometry was used to determine the presence of heavy metals such as Pb, Hg, Cd, and Ni (120). A low Ni concentration of 0.15 mg/kg was observed, in contrast to the findings of an earlier. In a separate study in Diyala, Iraq, in which a high concentration of 0.414 mg/kg of Ni was found in poultry (117). A lower Zn concentration of 20.72 mg/kg in chicken liver samples has been reported in Saudi Arabia (135), and a higher concentration of 100.87 mg/kg was observed in Turkiye. In previous studies, Cd concentrations of 0.159 mg/kg, 0.29 mg/kg, and 0.37 mg/kg had been observed in the liver in chickens in Iraq (107), Nigeria (115), and Iran (114), respectively. A Pb concentration of 0.28 mg/kg was observed in chicken liver samples, which is more than twice the limit of 0.1 mg/kg permitted by the Codex Alimentarius Commission. According to various studies, a low Pb concentration of 0.14 mg/kg has been observed in Saudi Arabia (117) and a higher Pb concentration of 0.171 mg/kg has been observed in Nigeria (115). In 40% of the samples, an Hg concentration of 0.11 0.08 mg/kg was found, which is above the FAO/WHO acceptable limit; this figure is three times greater than reported in previous Nigerian research (115).

### Histopathological changes in the kidneys in poultry

The kidneys, which are responsible for excreting poisonous substances, are the organs second-most severely impacted by Cd poisoning (136). When poultry are given Cd at a concentration of 50 mg/l in the drinking water, their kidneys have been found to develop congestion, with or without pinpoint hemorrhage (56). With administration of Cd to poultry at the same level in the drinking water, microscopic examinations of kidney tissues have revealed congested renal parenchyma, degeneration and desquamation of the tubule lining epithelium, hyaline masses, interstitial nephrosis, mononuclear cell infiltration, necrosis in the renal tubules, hypercellularity of glomeruli, and intracytoplasmic hyaline cast in the lumen (137). Cd-induced toxicity in the kidneys also causes changes in cell adhesion, autophagic responses, and cellular signaling cascades (54).

Additionally, histopathological changes in the kidney indicate necrotic lesions and eosinophilic intranuclear inclusion in epithelium cells of the renal tubules (138). Histopathological changes attributable to Hg accumulation in the kidneys have been found to include enlarged renal tubules, tubular hyalinization, fibrosis, fold increase in nucleosome content, increased levels of *malondialdehyde* (MDA), and decreased levels of intracellular glutathione (GSH) in the kidneys (139).

Finally, histopathological changes in the kidneys attributable to exposure to As include tubular fibrosis, enlargement of the renal tubules (140), severe hyalinization, increased renal MDA level, decreased renal SOD activity, decreased renal GSH-Px activity, and decreased CAT and GR activity (141).

### Histopathological changes in the liver in poultry

Cd is initially supplied to the liver through portal blood circulation, which is primarily associated with albumin, after which it is taken up by hepatocytes from the sinusoidal capillaries of the liver (63, 142). A higher Cd dosage results in increased hepatic cell size, hepatic cell destruction and necrosis, and significant infiltration of macrophages in the liver (143). Lower doses do not cause any notable alterations in poultry (144). Daily administration of Cd at 50 mg/l induces degenerative changes in the lymphocytes, macrophages, plasma cells, and hepatocytes, as well as producing swollen, fragile increases in the sinusoidal spaces and focal necrotic spots in livers. Cd causes primary hepatocellular injury, and thus ischemia is induced due to endothelial cell damage (145). Acute Cd exposure results in secondary liver injury due to the stimulation of Kupffer cells, eliciting a series of inflammatory events involving various types of liver cells and several inflammatory and cytotoxic mediators (146).

Absorbed Pb is accumulated in the liver, and Pb exposure may lead to histological abnormalities in the liver in poultry (147). After exposure to large doses of Pb, the livers of these animals have been found to exhibit abnormalities such as irregularity and dilatation of blood sinusoids, hepatic lipid vacuolization, vacuolization of other cells, hyalinization of the hepatocellular cytoplasm, hepatocyte necrosis, and severe sinusoid congestion (148). Additionally, Pb accumulation in the liver causes pinpoint hemorrhages and small necrotic foci (149).

In terms of histopathological changes in the liver after exposure to Hg in poultry, the sinusoids and central veins are dilated, hepatic cells show hypertrophy, and karyolytic and pycnotic cells are not prominent (150, 151). Finally, histopathological changes in the liver attributable to As accumulation include decreased GSH levels, increased hepatic MDA levels, decreased hepatic SOD activity, and decreased activity of CAT, GR, and GSH-Px (152).

### Histopathological changes in brain tissues in poultry

Histopathological changes in the brain after As poisoning in poultry include vacuolization and severe bleeding, which ultimately causes neuronal cell damage (153), lesions in the brain, mitochondrial swelling, and infiltration into glial cells (154).

### Histopathological changes in the reproductive system in poultry

The blood–testis barrier, certain seminiferous tubules, and the basement membrane have been found to undergo damage in poultry (155). As a result of Pb deposition, spermatogenic cells have been found to be organized erratically and to produce more spermatogonium, and the spermatogenic tubes are distorted (156).

## Conclusion

It can be concluded that worldwide heavy metal toxicity in poultry ranges from 2.1 to 3.4%. Chronic exposure to the heavy metals discussed here (i.e., As, Cd, Pb, and Hg) leads to their accumulation in various organs of the body; however, Cd accumulates at the highest concentrations, followed by As, Pb, and Hg in decreasing order. Various organs in poultry are affected by these heavy metals, with the sequence of impact beginning with the liver and continuing down to the kidneys, brain, and reproductive system. Overproduction of these heavy metals leads to oxidative stress in poultry. As a result of the accumulation of heavy metals, both gross and histopathological changes occur, leading to poor growth and production of multiple organs in poultry.

## Author contributions

AA worked on the development of this unique title of review, planned, designed, structured, wrote and reviewed the article.

## Conflict of interest

The author declares that the research was conducted in the absence of any commercial or financial relationships that could be construed as a potential conflict of interest.

## Publisher’s note

All claims expressed in this article are solely those of the authors and do not necessarily represent those of their affiliated organizations, or those of the publisher, the editors and the reviewers. Any product that may be evaluated in this article, or claim that may be made by its manufacturer, is not guaranteed or endorsed by the publisher.
